# Benefits of the Erector Spinae Plane Block before Cryoanalgesia in Children Undergoing Surgery for Funnel Chest Deformity

**DOI:** 10.3390/jpm13121696

**Published:** 2023-12-09

**Authors:** Sławomir Zacha, Konrad Jarosz, Karolina Kokot, Jarosław Biłas, Karolina Skonieczna-Żydecka, Sylwester Gerus, Klaudyna Kojder, Jowita Biernawska

**Affiliations:** 1 Department of Paediatric Orthopaedics and Musculoskeletal Oncology, Pomeranian Medical University, 71-252 Szczecin, Poland; s.zacha@spsk1.szn.pl (S.Z.); j.bilas@spsk1.szn.pl (J.B.); 2 Department of Clinical Nursing, Pomeranian Medical University, 70-204 Szczecin, Poland; 3 Department of Anaesthesiology and Intensive Therapy, Pomeranian Medical University, 71-252 Szczecin, Poland; k.kokot@spsk1.szn.pl (K.K.); klaudyna.kojder@pum.edu.pl (K.K.); jowita.biernawska@pum.edu.pl (J.B.); 4 Department of Biochemical Science, Faculty of Health Sciences, Pomeranian Medical University, 70-204 Szczecin, Poland; karolina.skonieczna.zydecka@pum.edu.pl; 5 Department of Paediatric Surgery and Urology, Wroclaw Medical University, 50-367 Wrocław, Poland; sylwester.gerus@gmail.com

**Keywords:** erector spinae plane block, cryoanalgesia, acute pain, funnel chest, Nuss surgery

## Abstract

Thoracic surgery causes significant pain despite standard multimodal analgesia. Intraoperative cryoanalgesia may be a solution. The onset of the clinical effect of cryoanalgesia can take 12–36 h. The addition of a regional anaesthesia before the cryoanalgesia procedure can enable analgesic protection for the patient during this period. The main aim of the study was to evaluate the benefits of the erector spinae plane (ESP) block prior to Nuss surgery. The ‘control’ group consisted of 10 teenagers who underwent cryoablation together with intravenous multimodal analgesia according to the standard protocol. The ‘intervention’ group included 26 teenage patients who additionally received an erector spinae plane block before operation. Pain relief (*p* = 0.015), opioid use (*p* = 0.009), independent physical activity and rehabilitation (*p* = 0.020) were faster in the intervention group. No features of local anaesthetic drug toxicity or complications of the ESP block were observed. The bilateral ESP block together with intraoperative intercostal nerve cryoablation performed prior to Nuss correction of funnel chest were more effective in terms of pain control.

## 1. Introduction

A funnel-shaped anterior chest wall is due to a congenital deformity of the sternum and ribs [1]. It results from a disorder of intramedullary ossification. The deformity is not only a cosmetic defect causing significantly reduced self-esteem in patients, but can also negatively affect their cardiopulmonary function. There is increasing evidence to suggest that pectus excavatum is indeed associated with abnormal biventricular systolic function at rest, decreased exercise capacity and decreased pulmonary function [1,2]. The Haller index using chest computer tomography is the most useful predictor of impairment. The Haller index is based on the ratio of the widest dimension of the chest to the smallest distance between the sternum and the spine, with the normal value being 2.5. The indication for surgery is a value above 3.2 [2]. Age and gender significantly influence cardiopulmonary function, with older patients (18 years and above) being more symptomatic. Minimally invasive Nuss surgery using thoracoscopy is currently the treatment of choice [2].

Thoracic surgery causes severe pain that persists for several weeks after surgery. The results of clinical studies confirmed that patients who experienced unacceptable acute postoperative pain and who suffered severe acute complications because of pain are more likely to report persistent pain at thirty days and one year because of dysregulated inflammatory responses [3,4]. The risk of consolidation into chronic pain is more than thirty per cent [5]. The current standard treatment for acute pain relief and the prevention of chronic pain development is multimodal analgesia, involving the simultaneous use of analgesics with different mechanisms of action, as well as different techniques of administration [6]. In view of the complex innervation of the chest wall, there is no consensus on a single effective regional method for pain relief after chest wall correction. The commonly chosen techniques for regional analgesia are thoracic epidural anaesthesia, paravertebral blocks, intrapleural blocks, fascial blocks of the dorsal extensor muscle compartment (erector spinae plane or ESP blocks) or multilevel intercostal nerve blocks [7]. Regional analgesia is necessary for patient care as measured by lower pain scores, a reduction in opioid use, faster mobilisation and independence, an improvement in breathing and sleeping, and a reduction in morbidity and mortality [8]. A single-shot approach or continuous anaesthesia via catheter implantation can be chosen.

In Poland and many countries, analgesia is most often administered using a catheter placed in the thoracic epidural space [9]. This allows the conduction of the branches of the thoracic spinal nerves to be blocked at multiple levels. Depending on the height of the block and the concentration of the local anaesthetic drug, the duration of the block can be controlled. The catheter failure rate is 16% in the thoracic epidural space [10]. A second popular method is to use a bilateral erector spinae plane (ESP) block and deposit the local anaesthetic drug in the intercostal space between the dorsal extensor muscle and the intercostal muscles [9]. Due to anatomical reasons (the thickness of muscle’s epimysium and the size of needle’s outer diameter), the precise injection of fascial plane blocks could be a challenge [10]. Moreover, improper administration of a fascial plane block may cause myotoxicity due to the local anaesthetic. On the other hand, ultrasound-guided fascial plane blocks of the chest wall showed significant benefits in thoracic surgery [11]. Several meta-analyses show a reduction in opioid use and pain for 24 h when fascial plane blocks were introduced [12]. The use of regional anaesthesia in perioperative care may reduce the risk of chronic post-surgical pain [13]. Bupivacaine or ropivacaine are usually chosen as a long-acting local anaesthetic. However, when the catheter is removed and regional analgesia is discontinued or when the drug’s duration of action ends, severe pain returns. The consequence is the risk of adverse effects of pharmacotherapy and local complications resulting from the catheter insertion and maintenance [11]. This may lead to prolonged hospitalisation and delayed rehabilitation and thus increases the total cost of treatment.

In view of the fact that multimodal analgesia via pharmacotherapy is insufficient, adjunctive methods are being sought to improve patient comfort, safety and recovery.

The use of low temperatures for pain relief has been known since the time of Hippocrates [14]. The modern version of ‘nerve freezing’ uses the Joules–Thomson phenomenon [15]. Low temperature causes the formation of ice crystals and an osmotic shift of water from the cell interior to the interstitium with temporary structural and functional destruction. Adjacent tissues, including nerves, are transiently damaged, temporarily blocking the conduction of pain sensations. Histological studies have shown that cryosectioning causes damage to axons while maintaining integrity within the perineurium and epineurium [16]. The intact innervation (endoneurium), blood vessels and myelin sheath, together with cytokines and growth factors remaining in the nerve environment, condition the nerve to rebuild along the Key–Retzius sheath. This prevents the formation of a neuroma. The structural and functional regeneration of the axons occurs within a few to several weeks. The local autoimmune effect of cryoablation results in the elimination of pathological receptor sites. There is minimal risk of pain consolidating into a chronic form. The first report of the use of cryoanalgesia for the Nuss procedure was published in 2016 [17]. The beneficial effect of cryoablation thus ensures a shorter duration of the usage of strong analgesic drugs and thus a reduction in their side effects. A faster return of independence and more effective postoperative rehabilitation result in shorter hospitalisation. To date, the results of clinical studies support these hypotheses, but these reports only involve a small number of patients [14,15,16,17,18].

The timing of the onset of the clinical analgesic effect of intercostal nerve cryotherapy has not been clearly established. Studies have suggested that the onset of action is not immediate [18]. Therefore, methods to bridge analgesia until the onset of action of intraoperative cryoanalgesia are being sought. Intraoperative cryoablation performed during Nuss surgery has the advantage of being able to precisely localise the neurovascular bundle. This does, however, necessitate pain protection for the patient for 1–2 days after the operation until the full effect of cryoablation is achieved. An alternative method may be percutaneous cryoanalgesia performed in advance, i.e., 12–48 h before surgery [19,20]. However, this procedure is associated with the need for additional sedation or general anaesthesia, indirect visualisation of the nerves with ultrasound and prolongation of the patient’s hospital stay.

The Cryo-S Painless apparatus from the Polish company Metrum Cryoflex, together with the A-30/300/PEA/R/RF cryogenic probe, was developed to perform intraoperative cryoanalgesia during thoracoscopy [21]. To date, the procedure of freezing nerves during surgery has been used in the USA and several European countries. In Poland, intraoperative cryoanalgesia of the intercostal nerves during Nuss surgery in children was performed for the first time in May 2022 at the Department of Paediatric Orthopaedics and Musculoskeletal Oncology of the Pomeranian Medical University in Szczecin [22].

The aim of this study was to evaluate the benefits of performing a ‘single shot’ ESP block in teenagers with funnel chest prior to minimally invasive Nuss correction with intraoperative cryoablation on their postoperative recovery.

## 2. Materials and Methods

### 2.1. Study Design and Patients

This was a prospective, non-randomised, single-centre pilot study. The general concept of the perioperative management was similar to our previous study [22]. We recruited 36 patients under 18 years of age undergoing minimally invasive elective Nuss surgery for funnel-shaped deformity of the anterior chest wall. The exclusion criteria were:

- Chest deformity other than funnel-shaped;
- Advanced chronic respiratory or circulatory failure;
- Emergency surgery or reoperation;
- History of thoracotomy or thoracic surgery;
- Mental impairment precluding communication with the patient or lack of consent for cryoanalgesia or regional analgesia;
- History of allergy to local anaesthetics;
- History of use of medication for chronic pain [22].

All study patients’ legal guardians (and they themselves if they were >16 years old) gave written informed consent to participate in the study. Our protocol was registered under NCT number 05589246 in the clinicaltrials.gov registry. Bioethics committee approval number KB-006/43/2022 was obtained from Pomeranian Medical University, Szczecin, Poland.

The ‘intervention’ group: 26 patients (mean age 15 years, range 11–17 years), which included 22 boys. These patients, after the induction of general anaesthesia, received regional anaesthesia in the form of a block of the dorsal extensor muscle compartment. An intraoperative nerve-freezing procedure was used along with multimodal analgesia using the same protocol as in the control group.

The ‘control’ group: 10 patients (median age 15 years, range 12–17 years), including 9 boys. These patients did not receive a regional block due to a refusal of or allergy to local anaesthetics. Intraoperative cryoablation was used along with multimodal analgesia according to the standard protocol.

### 2.2. Preparation for Surgery

The Haller index (defined as the ratio of the widest dimension of the chest to the smallest distance between the sternum and the spine) was determined by means of chest computer tomography [23]. A value above 3.2 is an indication for surgery. After scheduling the surgery, each patient had an outpatient anaesthetic consultation for qualification for general and regional anaesthesia.

On the day of surgery, the patient received metamizole (15 mg/kg for children under 50 kg or 1g orally if weight was over 50 kg), along with a carbohydrate-rich fluid (Preop, Nutricia, Poland) given orally 2 h before surgery [22].

### 2.3. Anaesthesia

The general concept of the intraoperative management was similar to our previous study [22]. All patients underwent general anaesthesia with double-lumen tube intubation, fibreoptic control of tube position and mechanical ventilation using the Primus device (Drager). After the induction of general anaesthesia (fentanyl 4 mcg/kg, propofol 3 mg/kg, ketamine 0.5 mg/kg, rocuronium 0.6 mg/kg, sevoflurane 1.0–1.3 minimal alveolar concentration), patients were administered the drugs according to multimodal analgesia principles. The drugs administered intravenously during the procedure were fentanyl (2 mcg/kg), paracetamol (15 mg/kg for children under 50 kg or 1g intravenously if weight was over 50 kg), ibuprofen (10mg/kg for children under 40 kg or 400 mg intravenously if weight was over 40 kg), magnesium sulphate (50 mg/kg via intravenous infusion), dexamethasone (0.15 mg/kg), ondansetron (0.15 mg/kg) [22].

After the induction of general anaesthesia in the intervention group, an ESP block was performed using an ultrasound (according to NYSORA’s Nerve Blocks protocol, NY, USA) and 0.25% bupivacaine at 0.3 mL/kg per side (no more than 20 mL per side) [22].

The maintenance of general anaesthesia with sevoflurane and fentanyl was carried out according to the standard protocol. The selective ventilation of one lung was carried out for the duration of cryoablation. The reversal of neuromuscular blockade with sugammadex 2 mg/kg and emergence were conducted according to the standard protocol. Approx. 20 min before the end of the procedure, an opioid bolus was administered, consisting of morphine <12 year or oxycodone >12 year, at a dose of 100 mcg/kg, and then continued with an infusion of 10–40 mcg/kg/h on an infusion pump for 24–36 h, depending on the intensity of pain as assessed by the numerical rating scale (NRS) [22].

### 2.4. Surgery

The surgical procedure was described in our article previously [22]. The qualification for surgery was based on a physical examination and CT scan. All the patients were operated on due to their diagnosis of funnel-shaped thoracic deformity. The appropriate length of the plate (Micromed, Poland) was selected, and the plate was dogged according to the size and shape of the thorax. The place of the insertion of the plate into the pleural cavity was marked with a marker in the most prominent intercostal spaces on both sides of the thoracic cavity. Bilateral skin incisions were made in the anterior axillary lines transverse to the long axis of the body. Two ports for thoracoscopy were then made in the mastoid lines approximately 4 cm below the skin incisions, and a plate was passed under the sternum.

Intraoperatively, an intercostal nerve cryoablation procedure was performed using the Cryo-S Painless device. The A-30/300/PEA/R/RF probe is dedicated to intraoperative use. The probe was inserted into the pleural cavity in the same intercostal space as the previously inserted plate, using the skin incision made. Adequate visualisation conditions were ensured by the ongoing ventilation of one lung on the contralateral side. Routinely, the cryoablation procedure was performed at six levels—usually Th4–Th10. The correspondingly curved tip of the probe was applied below the lower edge of the rib approximately 2 cm lateral to the ends of the transverse processes. The duration of a single cryoablation application was 2 min per intercostal nerve. A similar procedure was performed symmetrically on the opposite side. In total, the cryoanalgesia procedure lasted approximately 25–30 min.

The final stage of the procedure was to rotate the previously inserted plate 180 degrees. During this manoeuvre, the rotated plate lifted the sternum together with the adjacent deformed parts of the ribs. For large deformities, a direct lift was applied behind the sternum and 2 correction plates were used. Both ends of the plate rested on the ribs in the anterior axillary lines. To prevent secondary displacement of the implant, stabilising plates were placed unilaterally. In addition, both ends of the plate were fixed to the ribs with an insoluble thread. Before stratified suturing of the surgical accesses, a thoracoscopic inspection of the plate passage site under the sternum was performed to exclude bleeding [22].

### 2.5. Postoperative Course

The postoperative course was described in our previous study [22]. After surgery, all patients were observed in the postoperative room for at least 24 h. In addition to the assessment of vital signs, a protocol was followed for the assessment of pain intensity according to the NRS every 1 h for 24 h and then every 8 h until hospital discharge, indicating the type, doses and route of medication administered, and the occurrence of side effects and complications. Medications in the postoperative period were administered at fixed intervals: paracetamol (15 mg/kg under 50 kg or 1 g if weight was over 50 kg) orally or intravenously every 6 h; metamizole (15 mg/kg under 50 kg or 1 g if weight was over 50 kg) orally or intravenously every 6 h; ibuprofen (10 mg/kg under 40 kg or 400 mg if weight exceeded 40 kg) orally or intravenously every 8 h; and morphine infusion 10–40 mcg/kg/h on infusion pump and boluses max 0.1 mg/kg every 4 h if NRS at rest exceeded 3 points or on effort exceeded 6 points. Intravenous opioid infusion and the conversion to oral treatment were determined according to individual daily requirements. The administration of anaesthesia in the study is depicted in Table 1.

### 2.6. Outcomes

After discharge from the hospital, the first scheduled follow-up took place 10–14 days after surgery. The effect of the surgery, the location and intensity of pain and chest wall sensory disturbances were assessed.

The adverse effects of pharmacotherapy were defined as nausea, vomiting, difficulty breathing, pruritus, constipation, urinary retention, dizziness, drowsiness preventing rehabilitation, apnoea, hypotension depending on age-specific physiological values, bradycardia and drop in saturation of oxygen <90%.

The complications of the cryoablation procedure were defined as neuropathic pain, hypersensitivity, paresthesis and hypersensitivity of the operated area.

The complications of Nuss surgery were defined as respiratory and circulatory failure, pneumothorax requiring drainage, haematoma, surgical site infection, pleural effusion or abscess, pericarditis, bar dislocation requiring reoperation, pain impossible to relieve by standard means, pneumonia, heart perforation and death.

We evaluated the following outcomes:

- The acute pain intensity (maximum) on the first day after surgery—measured every 1 h for 24 h using the NRS numerical scale (0–10 points);
- The duration of the requirement for intravenous opioid use (days after surgery);
- The quality of postoperative rehabilitation in terms of correctness of exercises and achievement of independence of movement in daily activities (days after surgery) based on a self-assessment;
- The duration of surgery from skin incision to skin suture (minutes);
- The length of stay (LOS) in a hospital (days);
- The occurrence of adverse reactions to pharmacotherapy and anaesthesia;
- The occurrence of complications after cryoablation and Nuss surgery [22].

**Figure 1:**
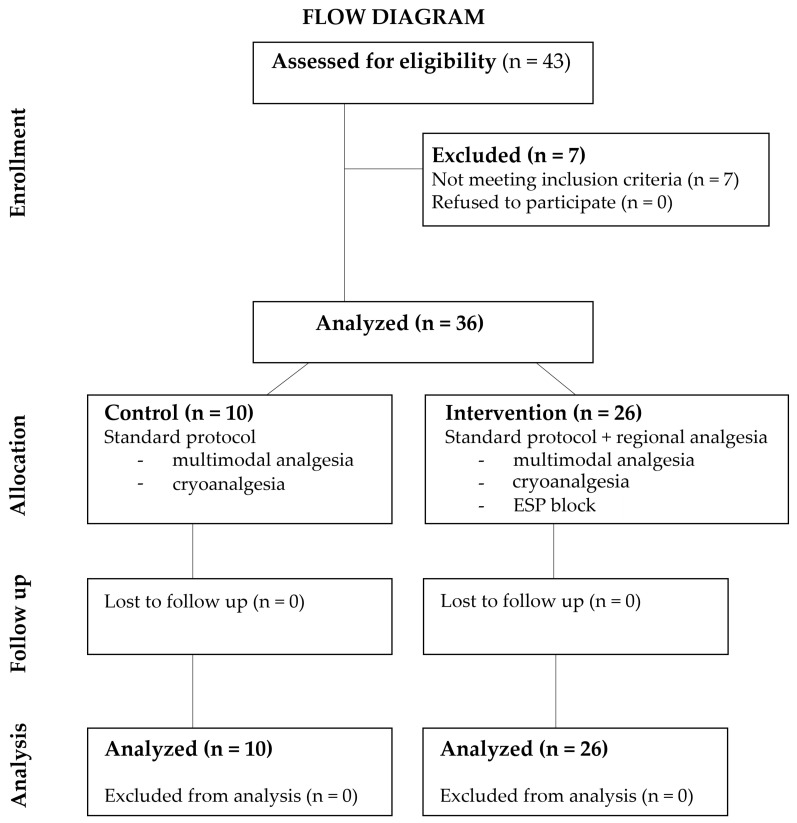
Flow diagram. Legend: ESP—erector spinae plane.

The flow diagram provided in Figure 1 illustrates the flow of patients throughout the study.

### 2.7. Statistics

The statistical analyses were performed in MedCalc statistical software, version 20.218 (Ostend, Belgium). We considered a two-sided *p* < 0.05 as a statistically significant difference. The Shapiro–Wilk test was used to verify the normality of the distribution of continuous data. Consequently, these were expressed as medians ± interquartile ranges (IQRs). Categorical data were expressed as numbers (percentages) and compared using chi-square or Fisher’s exact test. To assess the differences in continuous variables between groups, the Mann–Whitney test was adopted.

## 3. Results

### 3.1. Patients Characteristics

There were 22 (85%) and 9 boys (90%) in the intervention and control groups, respectively (*p* = 0.17). In each group, one case of asthma was declared (*p* = 0.51). No other co-morbidities were reported. The groups were similar in terms of demographics, age, BMI, Haller index scores and American Society of Anaesthesiology (ASA) scale score (Table 2).

### 3.2. Treatment Efficacy

**Figure 2:**
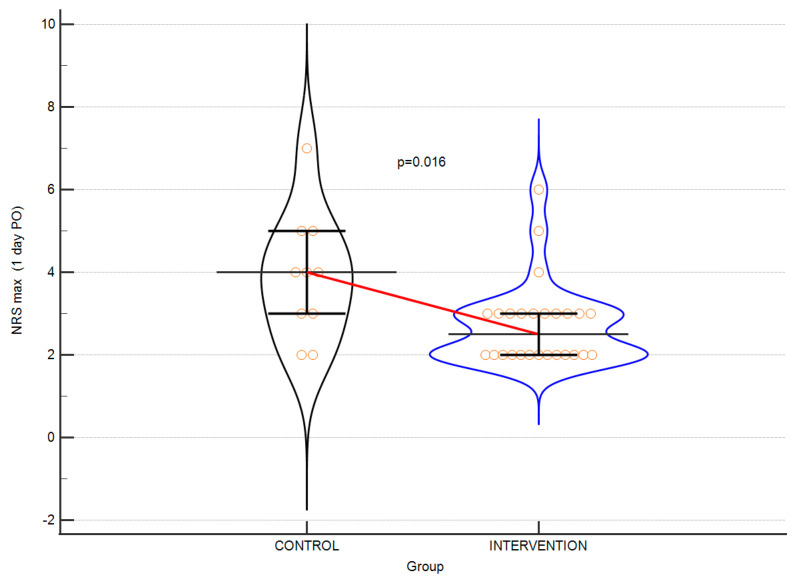
Violin plots depicting the difference in NRS scores at postoperative day 1. Horizontal line connects medians. Error bars indicate IQRs.

A comparative analysis of the parameters analysed in the perioperative period showed statistically significant differences in pain intensity on the first postoperative day (Figure 2) and the requirement for intravenous opioid use in favour of the intervention. The time of surgery and LOS were similar in both groups. The results are shown in Table 3.

**Figure 3:**
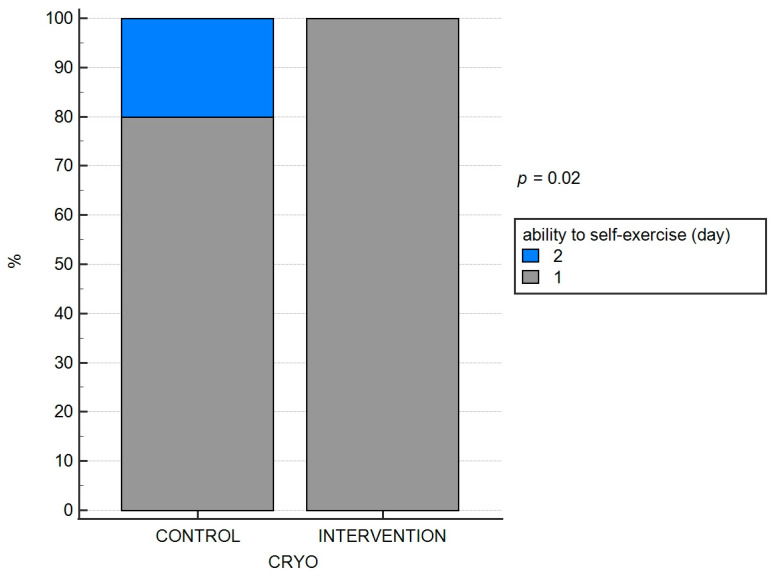
The efficacy of cryo-intervention on the ability to perform exercise in each position and (**B**) change position.

**Figure 4:**
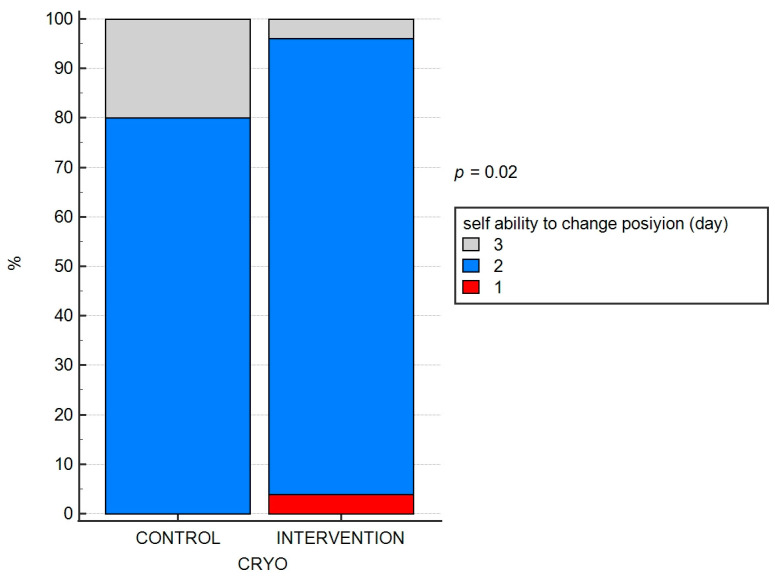
The efficacy of cryo-intervention on the ability to change position.

The ability to change body position independently and to perform exercises in any position (independence) on the second postoperative day was achieved by eight of the ten patients in the ‘control’ group and, by the third postoperative day, by the remaining two patients. Similarly, in the ‘intervention’ group, 1 patient achieved this milestone on the first postoperative day, 24 patients achieved it on the second postoperative day and 1 patient on the third postoperative day. These differences were significant (*p* = 0.020; both). The results are depicted in Figure 3 and Figure 4.

### 3.3. Adverse Effects

The adverse effects after pharmacotherapy included postoperative nausea and vomiting in both groups. No features of local anaesthetic drug toxicity (LAST) nor complications of the ESP blockade were observed, as evidenced with the use of the chi2 test.

The complications after the Nuss operation included a loose plate with the need for revision in one case and mild pneumothorax in the remaining cases. At discharge from the hospital, in one case from each group, a transient hypersensitivity on the chest was observed. The differences were not significant (*p* = 0.283).

No early complications of cryoanalgesia were observed. Fourteen and thirty days after surgery, there were no predefined complications of the regional block. In four patients, hyperalgesia was observed, and in six patients, hypoalgesia was observed. These disturbances of sensation were well tolerated. No case of neuropathic pain was observed. For details, see Table 4.

## 4. Discussion

The results of this study demonstrate the benefits of performing a ‘single shot’ ESP block prior to minimally invasive Nuss surgery together with intraoperative intercostal nerve cryoanalgesia in patients with a diagnosis of funnel-shaped thoracic deformity. This is the first study in which the effectiveness of ESP blockades as a method in combination with intraoperative intercostal nerve cryoablation was confirmed in terms of less intensity of acute pain after surgery and a shorter demand for intravenous opioid treatment. This enabled a quicker achievement of motor independence and the correct execution of exercises performed during rehabilitation after surgery. Better pain control and the reduction of the sedative effect of opioids helped the patients to both rehabilitate effectively and return to independence faster. At the same time, the additional intervention in the form of an ESP block did not significantly increase the whole procedure time.

A major challenge for the thoracic surgery team is the control of severe pain in the postoperative period. Poor control of postoperative pain has consequences in terms of a high risk of extended pain after the procedure, delayed postoperative rehabilitation, longer hospitalisation, a lack of patient and family satisfaction and higher costs of hospitalisation [2].

To date, the use of recommended intravenous pharmacotherapy and regional multimodal analgesia has not effectively relieved pain [6]. The solution to this problem was the addition of an intercostal nerve cryotherapy procedure. This procedure is currently recommended [18,19,20,21,22]. The results from clinical observations have shown that the peak of the therapeutic effect of cryotherapy is not immediate. To date, the optimal time to perform cryoablation before Nuss surgery has not been established [17,18,19]. The authors of the present study observed that, in their patients, the use of cryoablation together with the performance of a bilateral ESP blockade with long-acting local anaesthetics provides more effective pain relief than isolated intravenous multimodal analgesia in combination with cryoablation. The effects of the ‘single shot’ ESP blockade lasted for approximately 16–24 h after surgery.

There is much favourable evidence in the literature for the addition of a cryoablation procedure to intravenous multimodal analgesia. Although the patient groups studied were small and the type of regional anaesthesia protocol used during multimodal analgesia varied each time, the authors noticed that significantly better results were obtained when a cryoablation procedure was added to the standard protocol [24,25,26,27].

In terms of the influence on the efficacy of acute pain relief and reduction in the total dose of intravenous opioids, the results of our study are similar to those reported in the literature to date [28,29].

The ESP ‘single shot’ blockade has the beneficial effect of reducing the amount of opioids needed. It resulted in a reduction in the total dose of opioids and the occurrence of adverse effects after pharmacotherapy [30,31]. Cryoablation performed together with the ‘single shot’ ESP blockade is therefore a safer option compared to keeping the catheter to continue regional analgesia for several days.

One may consider the possibility of using cryoablation both intraoperatively and percutaneously, even a few days before the Nuss procedure. The question arises as to which method carries more advantages. The intraoperative technique of intercostal nerve cryoanalgesia during thoracoscopy has the undoubted advantages of direct contact and visual control. The disadvantage is the short time between the cryoanalgesia and surgery. The percutaneous cryoanalgesia makes it possible to perform it before surgery at any time in advance. However, it involves additional general anaesthesia for paediatric patients and suboptimal control of the probe position through indirect visualisation (ultrasound) [19,20]. Our results on postoperative pain intensity, the duration of intravenous opioids and the effectiveness of postoperative rehabilitation showed that preoperative regional analgesia (bilateral ESP block) followed by intraoperative intercostal nerve cryoablation may be the best option.

Other debatable issues are the complications of the procedures used: the toxicity of local anaesthetic drugs, some anatomical considerations about fascial plane blocks, the risk of developing a neuroma, the time taken for full nerve regeneration and the incidence of neuropathic pain. The ultrasound-guided ESP block and intraoperative intercostal nerve cryoablation procedures are safe. The following complications have been reported in the literature: persistent neuropathic pain, hypersensitivity, transient pain in the operated area, bleeding from the wound, bleeding from deep structures, haematoma, lung injury and clinically significant pneumothorax [32,33,34,35,36,37]. The exact statistics on the incidence of complications are not known; in total, they probably account for less than 1% of cases. In our study, no complications of the regional block were observed. We noticed the occurrence of minor transient hypo- and hypersensitivities on the chest.

## 5. Conclusions

A bilateral ESP block along with intraoperative intercostal nerve cryoablation performed prior to Nuss correction of a funnel chest deformation was more effective than the routinely used intravenous multimodal analgesia along with the cryoablation procedure. This makes it possible to reduce the duration of opioid use and improve the quality of postoperative rehabilitation.

## Figures and Tables

**Table 1:** The anaesthesia protocol used in this study.

Control	Intervention
Preparation: pre-emptive analgesia	
metamizole orally carbohydrate-rich fluid antibiotic prophylaxis	metamizole orally carbohydrate-rich fluid antibiotic prophylaxis
Induction of general anaesthesia under standard monitoring	
fentanyl, propofol, ketamine, rocuronium sevoflurane DLT, fibroscopy	fentanyl, propofol, ketamine, rocuronium sevoflurane DLT, fibroscopy
Maintenance of general anaesthesia	
sevoflurane, fentanyl, paracetamol, ibuprofen, magnesium sulphate, dexamethasone, ondansetron	sevoflurane, fentanyl, paracetamol, ibuprofen, magnesium sulphate, dexamethasone, ondansetron
-	ESP block
intraoperative cryoanalgesia with OLV	intraoperative cryoanalgesia with OLV
At the end of surgery	
sugammadex morphine or oxycodone	sugammadex morphine or oxycodone
Postoperative course: standard monitoring and regular pain assessment	
paracetamol, metamizole, ibuprofen i.v./orally morphine or oxycodone next: oxycodone with naloxone orally	paracetamol, metamizole, ibuprofen i.v./orally morphine or oxycodone next: oxycodone with naloxone orally

Legend: DLT—double-lumen tube; ESP—erector spinae plane; OLV—one lung ventilation; i.v.—intravenously.

**Table 2:** Demographic data and preoperative assessment of the study population.

Parameter	Control (*n* = 10)	Intervention (*n* = 26)	*p*
Age	15 (12–17)	15 (11–17)	0.77
BMI	18 (14–20)	18 (14–20)	0.46
ASA 1	9 (90)	25 (96)	0.47
Haller index	3.35 (3.2–3.9)	3.4 (3.2–3.9)	0.87

Legend: *n*—number; BMI—body mass index; ASA—American Society of Anaesthesiology scale score.

**Table 3:** The efficacy of intervention on parameters measured in perioperative period.

Parameter	Control (*n* = 10)	Intervention (*n* = 26)	*p*
Surgery time, median (IQR)	80 (60.0–92.5)	72.5 (65.0–90.0)	0.863
MAX NRS 1, median (IQR)	4. 0 (3.0–5.0)	2.5 (2.0–3.0)	0.015
Length of hospitalisation (days)	5 (50)	4 (15)	0.382
Discontinuation of opioids 1 POD, *n* (%)	3 (30)	20 (76.9)	0.009
Discontinuation of opioids 2 POD, *n* (%)	7 (70)	6 (23.1)	

Legend: *n*—number; MAX NRS 1—acute pain intensity value (maximum) on the first postoperative day according to the NRS scale; POD—postoperative day.

**Table 4:** Adverse events.

Parameter	Control (*n* = 10)	Intervention (*n* = 26)	*p*
Adverse effects after pharmacotherapy n (%)	2 (20)	3 (11)	0.516
Complications after Nuss surgery n (%)	5 (50)	8 (31)	0.283
Complications after anaesthesia (early)	0	0	n.e.

Legend: n.e.—not estimable.

## Data Availability

The raw data are available upon request from the corresponding author.
